# Dim Light at Night Disturbs Molecular Pathways of Lipid Metabolism

**DOI:** 10.3390/ijms21186919

**Published:** 2020-09-21

**Authors:** Monika Okuliarova, Valentina Sophia Rumanova, Katarina Stebelova, Michal Zeman

**Affiliations:** Department of Animal Physiology and Ethology, Faculty of Natural Sciences, Comenius University, Ilkovicova 6, 842 15 Bratislava, Slovakia; rumanovavalentina@gmail.com (V.S.R.); katarina.stebelova@uniba.sk (K.S.); michal.zeman@uniba.sk (M.Z.)

**Keywords:** chronodisruption, liver, steatosis, lipogenesis, fatty acids, glucose, insulin, leptin, nuclear receptors, circadian clocks

## Abstract

Dim light at night (dLAN) is associated with metabolic risk but the specific effects on lipid metabolism have only been evaluated to a limited extent. Therefore, to explore whether dLAN can compromise lipid metabolic homeostasis in healthy individuals, we exposed Wistar rats to dLAN (~2 lx) for 2 and 5 weeks and analyzed the main lipogenic pathways in the liver and epididymal fat pad, including the control mechanisms at the hormonal and molecular level. We found that dLAN promoted hepatic triacylglycerol accumulation, upregulated hepatic genes involved in de novo synthesis of fatty acids, and elevated glucose and fatty acid uptake. These observations were paralleled with suppressed fatty acid synthesis in the adipose tissue and altered plasma adipokine levels, indicating disturbed adipocyte metabolic function with a potential negative impact on liver metabolism. Moreover, dLAN-exposed rats displayed an elevated expression of two peroxisome proliferator-activated receptor family members (*Pparα* and *Pparγ*) in the liver and adipose tissue, suggesting the deregulation of important metabolic transcription factors. Together, our results demonstrate that an impaired balance of lipid biosynthetic pathways caused by dLAN can increase lipid storage in the liver, thereby accounting for a potential linking mechanism between dLAN and metabolic diseases.

## 1. Introduction

Hepatic lipid homeostasis is maintained through a balance among metabolic pathways controlling the handling of triacylglycerols (TAGs) and fatty acids in the liver [1]. This balance is a tightly regulated system of signaling and transcriptional networks, and its disruption can lead to excessive lipid accumulation that, in turn, may progress to nonalcoholic fatty liver disease (NAFLD) [1,2,3]. NAFLD is considered the most prevalent chronic liver disease worldwide and is usually associated with a spectrum of metabolic pathologies encompassed by metabolic syndrome [3]. Important molecular mechanisms driving hepatic steatosis include several contributing factors, such as impaired insulin signaling at the level of the adipose tissue and liver, elevated transport of fatty acids to the liver, and hepatic de novo lipogenesis [2,4]. Rate-limiting enzymes for de novo lipogenesis are acetyl-CoA carboxylase (ACC) and fatty acid synthase (FASN), which catalyze the first steps in the fatty acid synthesis [5]. Both are transcriptionally regulated by various nuclear receptors, including peroxisome proliferator-activated receptors (PPARs), which represent important regulators of lipid metabolic pathways in different body tissues [6].

A complex regulation of hepatic lipid metabolism is the result of crosstalk among metabolic hormones, energy/nutrient-sensing signals, and a circadian system [7]. The mammalian circadian system consists of a master clock in the hypothalamic suprachiasmatic nuclei and subordinate peripheral oscillators in other brain regions and peripheral tissues, including the liver and adipose tissue [7,8]. This internal timekeeping system generates and orchestrates circadian rhythms in daily physiology and behavior from the molecular level to the whole-body level in order to anticipate and coordinate periodic environmental changes and metabolic requirements [9]. The oscillations of the master clock are entrained by the main external time-giver, the 24-h light-dark (LD) cycle, and the processed signal is then transmitted via neuronal, hormonal, and behavioral pathways to the periphery [10].

Introducing light sources with high efficiency and a modern lifestyle have caused that the role of the LD cycle, as the dominant synchronizing agent for circadian rhythms, is increasingly compromised by artificial light at night [11,12]. This term usually includes various forms of excessive and inappropriately timed nocturnal light exposure in outdoor or indoor environments. Exposure to a low-illuminance level during the nighttime, experienced in light-polluted urban areas or resulting from the evening use of light-emitting devices, is known as dim light at night (dLAN) [13]. Although the day–night light intensity difference is not completely lost under dLAN conditions, dLAN has been shown to disturb the circadian clock function, as evidenced by suppressed nighttime melatonin levels [14,15], low-amplitude rhythms in locomotor activity and cardiovascular parameters [14,16], and an altered rhythmic variation of clock genes in the hypothalamus and the liver [16,17]. Circadian disruption in response to diverse environmental or endogenous perturbations can misalign metabolic homeostasis and thereby contribute to the development of metabolic disorders [18,19,20]. Accordingly, a growing number of epidemiological and experimental studies have reported an association between dLAN exposure and metabolic dysfunction, leading to an increased risk of obesity [21,22], type 2 diabetes mellitus [23], and cardiovascular disease [24,25]. Experiments in mice showed that dLAN (5 lx) exposure resulted in an impaired glucose tolerance and increased body mass, likely due to shifting the rhythm of food intake into daytime [26]. Thus, dLAN can impact metabolic homeostasis through disruption of behavioral rhythms, such as the sleep/wake and feeding cycles, which serve as the essential time-givers for peripheral oscillators, especially the liver [8]. Importantly, dLAN can amplify or accelerate detrimental metabolic effects in individuals with increased health risk and metabolic comorbidities [27,28]. Together, all these observations suggest that there is a need to reveal the mechanistic relationships between dLAN and metabolic consequences in more detail and at several control levels.

Although chronic dLAN exposure is often linked with negative metabolic consequences [29], the specific effects of dLAN on lipid metabolism have only been evaluated to a limited extent so far. In our previous study, we showed that dLAN enhanced hepatic TAG accumulation in spontaneously hypertensive rats, and this was associated with the upregulation of metabolic transcription factors *Pparα* and *Pparγ* in the liver and epididymal fat pad [27]. In the present study, we used Wistar rats to examine whether dLAN can compromise metabolic homeostasis and promote hepatic accumulation of lipids in healthy individuals following chronic exposure to low-intensity light (~2 lx) at night. To explore this hypothesis at the complex regulatory level, we analyzed (1) the amounts of hepatic TAGs, (2) the expression of metabolic genes functioning in the main lipid-handling pathways in the liver and adipose tissue, (3) the plasma levels of metabolic hormones and metabolites, and (4) the expression of important metabolic transcription factors of PPAR family. Moreover, we sampled the animals after 2 and 5 weeks of dLAN exposure in order to evaluate how dLAN-induced metabolic effects develop over time.

## 2. Results

### 2.1. dLAN Promotes Hepatic Accumulation of Lipids

**Figure 1:**
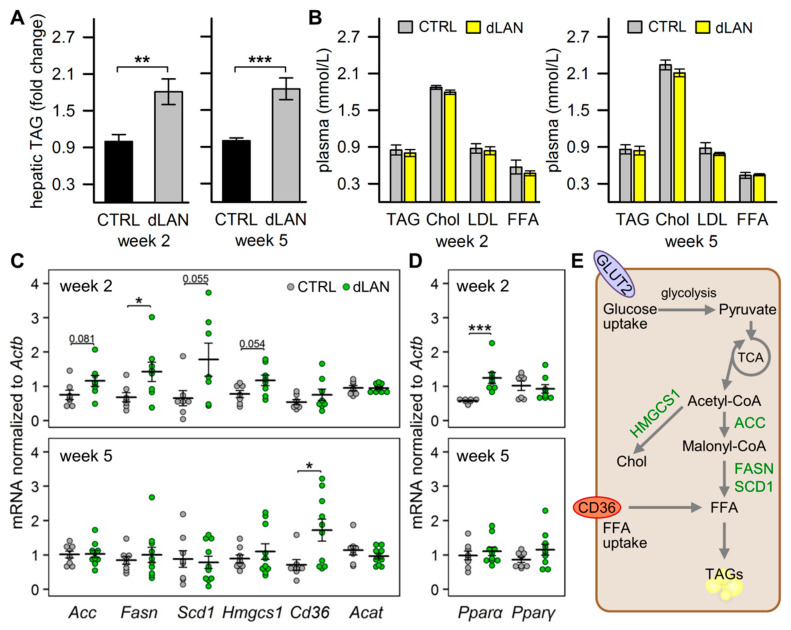
Dim light at night (dLAN) promotes accumulation of lipids in the liver. Rats were exposed to either the control LD regime (CTRL) or dLAN (~2 lx) for 2 and 5 weeks. (**A**) Hepatic triacylglycerol (TAG) levels normalized to CTRL groups. (**B**) Plasma levels of TAGs, total cholesterol (Chol), low-density lipoprotein (LDL) cholesterol, and free fatty acids (FFAs). Data represent the mean ± SEM. (**C**,**D**) Relative mRNA levels of hepatic lipogenic genes (**C**) and metabolic transcription factors (**D**). Data are shown as dot plots with the mean (thick horizontal lines) ± SEM (error bars). CTRL (*n* = 7–8 rats) and dLAN (*n* = 7–10 rats) groups were compared with the Student’s *t*-test or the Mann–Whitney U test. * *p* < 0.05, ** *p* < 0.01, *** *p* < 0.001. (**E**) Key metabolic steps in hepatic de novo lipogenesis, which can contribute to liver TAG accumulation under dLAN conditions.

Hepatic metabolic homeostasis is maintained in accordance with circadian oscillations; therefore, circadian misalignment induced by disturbed LD cycles can impair the homeostasis and result in ectopic accumulation of lipids in the liver [20,27,30]. In our study, we examined this link in rats, which were exposed to dLAN for 2 or 5 weeks. We found that rats exposed to dLAN displayed higher hepatic TAG levels compared to controls (CTRL) after both 2 and 5 weeks (Figure 1A). Next, we analyzed, whether these dLAN-induced effects are associated with an altered lipid profile in the circulation. The analyses showed that CTRL and dLAN-exposed rats did not differ in plasma TAG, cholesterol, low-density lipoprotein (LDL) cholesterol, and free fatty acid (FFA) levels after either 2 or 5 weeks (Figure 1B). Besides circulating FFAs, there are other important sources contributing to an excessive accumulation of lipids in the liver, including de novo lipogenesis [31]. Following 2 weeks of dLAN, rats showed higher hepatic *Fasn* mRNA levels and a trend towards increased expression of lipogenic genes *Acc*, stearoyl-CoA desaturase 1 (*Scd1*), and 3-hydroxy-3-methylglutaryl-CoA synthase 1 (*Hmgcs1*) compared to CTRL (Figure 1C). These changes were not detected in rats following 5 weeks of dLAN, but this exposure resulted in the upregulation of fatty acid translocase (*Cd36*) in the liver (Figure 1C). Therefore, both de novo synthesis of fatty acids and an increased hepatic fatty acid uptake can participate in excessive hepatic lipid accumulation under dLAN conditions (Figure 1E). An excess of fatty acids in the liver can be expected to redirect acetyl-CoA from the tricarboxylic acid cycle (TCA) to the synthesis of ketone bodies [5], but dLAN exposure did not affect hepatic mRNA levels of mitochondrial acetyl-CoA acetyltransferase (*Acat*) (Figure 1C), an enzyme involved in the ketogenic pathway.

Numerous genes involved in hepatic lipid metabolism are under the transcriptional control of PPARs [6]. Hepatic PPARα mainly activates genes required for the oxidation of fatty acids, but it also plays a role in the de novo synthesis of fatty acids [32]. In parallel with the upregulation of lipogenic genes after 2 weeks in dLAN, rats displayed higher hepatic mRNA levels of *Pparα* compared to controls, whereas no changes were detected in *Pparγ* (Figure 1D).

### 2.2. dLAN Increases Glucose Uptake into the Liver

**Figure 2:**
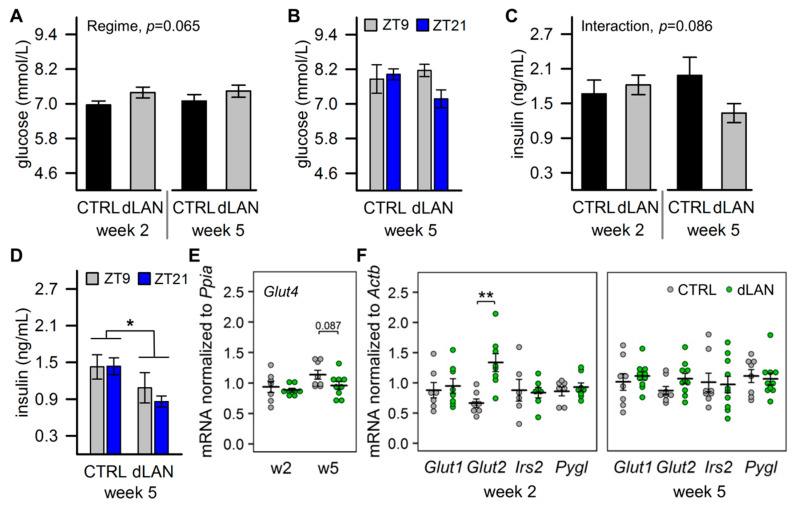
Dim light at night (dLAN) increases glucose uptake into the liver. (**A**–**D**) Plasma glucose (**A**,**B**) and insulin (**C**,**D**) levels in rats exposed to either the control LD regime (CTRL) or dLAN (~2 lx) for 2 (w2) and 5 (w5) weeks. Blood samples were collected either (**A**,**C**) after decapitation between Zeitgeber time 3 (ZT3) and ZT6 or (**B**,**D**) from a tail vein at ZT9 and ZT21. Data represent the mean ± SEM. CTRL (*n* = 7–8 rats) and dLAN (*n* = 7–10 rats) groups were compared with two-way ANOVA. * *p* < 0.05. (**E**,**F**) Relative mRNA levels of genes involved in glucose transport in the epididymal fat pad (**E**) and in glucose transport, insulin signaling, and glycogen pathway in the liver (**F**). Data are shown as dot plots with the mean (thick horizontal lines) ± SEM (error bars). CTRL and dLAN groups were compared with the Student’s *t*-test or the Mann–Whitney U test. ** *p* < 0.01.

Since de novo lipogenesis converts an excess of acetyl-CoA from carbohydrates to new fatty acids [4], we next analyzed glucose homeostasis in dLAN-exposed rats. Plasma glucose levels tended to be higher in rats exposed to dLAN compared to CTRL rats (Figure 2A), although this effect was not confirmed if, following 5 weeks in dLAN, animals were blood sampled 3 h before the end of the light (ZT9) and the dark/dim light (ZT21) phase, respectively (Figure 2B). In contrast, plasma insulin concentrations were reduced in rats after 5 weeks in dLAN compared to controls, and this difference was not dependent on the time of day (Figure 2C,D). Insulin regulates glucose uptake into the target tissues via insulin-dependent glucose transporter GLUT4. Thus, in line with reduced plasma insulin levels, we found a trend towards lower *Glut4* mRNA levels in the epididymal fat pad of dLAN-exposed rats compared to CTRL (Figure 2E). On the other hand, after 2 weeks, dLAN-exposed rats displayed higher hepatic mRNA levels of insulin-independent glucose transporter *Glut2* than CTRL rats (Figure 2F), indicating elevated glucose uptake into the liver. Hepatic gene expression of glucose transporter *Glut1* was not affected by dLAN, and no changes were found for insulin receptor substrate 2 (*Irs2*), a down-stream effector of insulin signaling, and glycogen phosphorylase L (*Pygl*), a rate-limiting enzyme catalyzing glycogen breakdown to glucose (Figure 2F). These observations suggest that dLAN-enhanced hepatic accumulation of TAGs is driven by elevated glucose uptake into the liver, and this was independent of insulin levels that were even reduced (Figure 1E).

### 2.3. dLAN Alters Lipid Metabolism in the White Adipose Tissue

**Figure 3:**
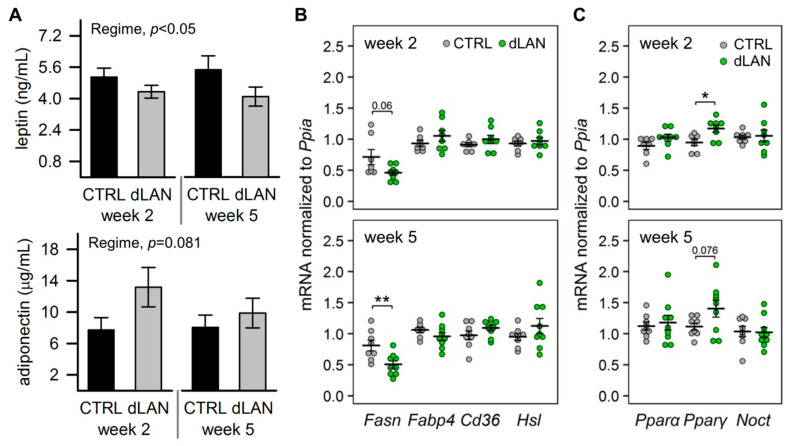
Dim light at night (dLAN) affects lipid metabolism in the white adipose tissue. (**A**) Plasma leptin and adiponectin levels in rats exposed to either the control LD regime (CTRL) or dLAN (~2 lx) for 2 and 5 weeks. Data represent the mean ± SEM. CTRL (*n* = 7–8 rats) and dLAN (*n* = 7–10 rats) groups were compared with two-way ANOVA. (**B**,**C**) Relative mRNA levels of lipogenic and lipolytic genes (**B**) and metabolic transcription and post-transcription regulators (**C**) in the epididymal fat pad. Data are shown as dot plots with the mean (thick horizontal lines) ± SEM (error bars). CTRL and dLAN groups were compared with the Student’s *t*-test. * *p* < 0.05, ** *p* < 0.01.

Based on enhanced hepatic lipogenesis, we also expected disturbances in lipid metabolism at the white adipose tissue level in dLAN-exposed rats. First, we assessed circulating levels of main adipokines, leptin, and adiponectin, which are produced in the adipose tissue and are involved in metabolic homeostasis [33]. We found lower leptin and a trend towards higher adiponectin in the circulation of rats exposed to dLAN compared to CTRL (Figure 3A). In contrast to the liver, in the epididymal fat pad, *Fasn* mRNA levels were lower in rats exposed to dLAN than in controls (Figure 3B), indicating suppressed de novo lipogenesis in the adipose tissue. This effect was not associated with the intracellular availability of fatty acids, since no differences were found in gene expression of *Cd36* and adipocyte fatty acid binding protein 4 (*Fabp4*) between CTRL and dLAN-exposed rats (Figure 3B). Similarly, mRNA levels of hormone-sensitive lipase (*Hsl*) (Figure 3B), a lipolytic enzyme, were not affected by dLAN.

PPARγ is mainly expressed in the adipose tissue, where it functions not only as an important adipogenic regulator but is also involved in several other lipid-handling pathways [34]. Following 2 weeks in dLAN, rats displayed higher *Pparγ* mRNA levels in the epididymal fat pad compared to controls, and the same trend was found after 5 weeks in dLAN (Figure 3C). On the other hand, no changes were recorded in gene expression of *Pparα* and nocturnin (*Noct*) (Figure 3C), a key post-transcriptional regulator of metabolic pathways and circadian rhythms [35].

Collectively, the data show that dLAN suppressed fatty acid synthesis in the epididymal fat pad and altered the adipokine balance in the circulation, indicating disturbed metabolic function in adipocytes with a potential impact on the liver.

### 2.4. dLAN Does not Affect Plasma Thyroid Hormone Levels

**Figure 4:**
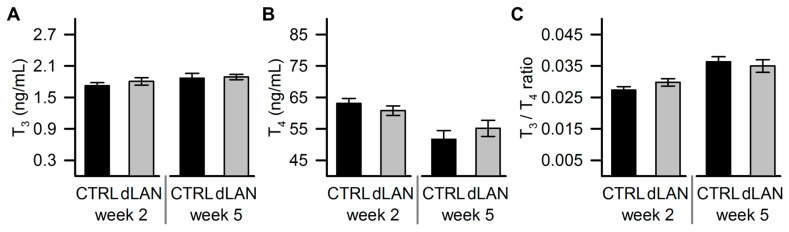
Dim light at night (dLAN) does not affect thyroid hormone levels in the circulation. (**A**) Plasma triiodothyronine (T_3_), (**B**) thyroxine (T_4_), and (**C**) T_3_/T_4_ ratio in rats exposed to either the control LD regime (CTRL) or dLAN (~2 lx) for 2 and 5 weeks. Data represent the mean ± SEM. CTRL (*n* = 7–8 rats) and dLAN (*n* = 7–10 rats) groups were compared with two-way ANOVA.

Next, we analyzed circulating levels of thyroid hormones, which are known to regulate metabolic pathways in the liver and adipose tissue [36]. Moreover, the hypothalamic–pituitary–thyroid axis is under circadian control, and disturbed lighting conditions have been shown to alter thyroid hormone levels [37]. However, we found no differences in plasma levels of triiodothyronine (T_3_), thyroxine (T_4_), and the T_3_/T_4_ ratio between CTRL and dLAN-exposed rats (Figure 4A–C), indicating that thyroid hormones were not primarily involved in metabolic effects caused by dLAN.

### 2.5. dLAN Does not Affect Body Mass

**Figure 5:**
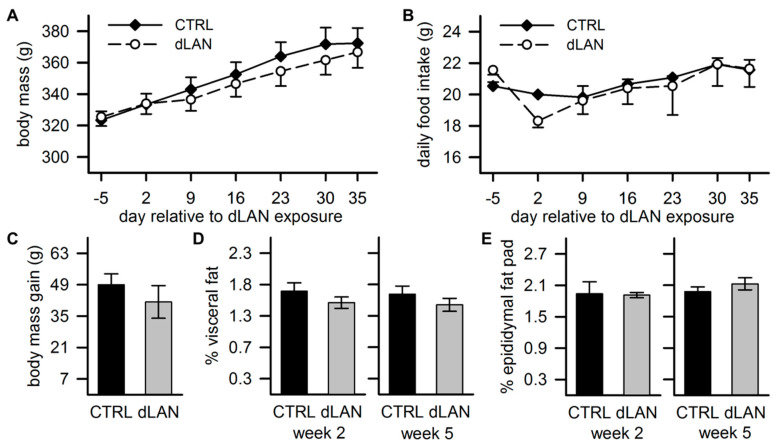
Dim light at night (dLAN) does not affect body mass. (**A**) Mean body mass and (**B**) daily food intake changes in rats exposed to either the control LD regime (CTRL) or dLAN (~2 lx) for 5 weeks. Data were analyzed with two-way repeated measures ANOVA. (**C**) Total body mass gain, (**D**) the percentage of visceral, and (**E**) epididymal fat pad analyzed in rats after 2 and 5 weeks in CTRL or dLAN regime. CTRL (*n* = 7–8 rats) and dLAN (*n* = 8–10 rats) groups were compared with the Student’s *t*-test. Data represent the mean ± SEM.

Previous studies in Swiss-Webster mice reported that dLAN exposure can lead to increased body mass [26], but this affect was not found in rats [15,16]. In our study, body mass increased over the 5 weeks of the experiment, but no differences between CTRL and dLAN-exposed rats were recorded, either in body mass (Figure 5A) or total body mass gain (Figure 5C). These findings were in line with daily food intake changes over the experiment, which did not differ between CTRL and dLAN-exposed rats (Figure 5B). Moreover, 2 and 5 weeks of dLAN did not affect the percentage of visceral fat (Figure 5D) and epididymal fat pad mass (Figure 5E). Therefore, dLAN-induced changes in metabolic homeostasis were probably independent of changes in body mass and total food intake.

## 3. Discussion

Excessive exposure to light at night is a globally widespread phenomenon of a modern society. During recent years, evidence has been accumulating that such disturbances of natural LD cycles can impose potential health risks, especially in association with metabolic dysfunction [29,38]. Therefore, there is an urgent need to elucidate the underlying molecular mechanisms linking dLAN with metabolic disorders. Here, we focused on lipid homeostasis and explored to what extent it is sensitive to dLAN exposure in healthy Wistar rats. We found that nocturnal exposure to low-intensity light promoted hepatic TAG accumulation, which was associated with enhanced de novo synthesis of fatty acids and elevated glucose and fatty acid uptake into the liver. These observations were paralleled with suppressed fatty acid synthesis in the adipose tissue and deregulated plasma adipokine levels, indicating disturbed metabolic function in adipocytes with a potential impact on the liver. Moreover, our data showed that transcriptional control of these dLAN-induced metabolic effects was linked with the upregulation of important metabolic transcription factors, *Pparα* and *Pparγ*.

The ectopic accumulation of lipids in the liver has been experimentally demonstrated following a chronic exposure to disrupted LD cycles, especially by phase shifts [20,39] and constant light [30]. The same effect resulted from dLAN in a rat model of essential hypertension [27] and here, we evidenced that 5-week dLAN exposure can trigger abnormal hepatic lipid accumulation in healthy animals. At the molecular level, we found upregulation of the main lipogenic genes, such as *Fasn*, *Acc*, and *Scd1*, suggesting that hepatic TAG storage in dLAN-exposed rats is driven by the elevated de novo synthesis of fatty acids in the liver. Interestingly, we found that this pathway was especially upregulated 2 weeks after dLAN exposure, indicating that other processes participated in excessive lipid storage. In line with this notion, 5 weeks of dLAN elevated hepatic *Cd36* expression, which implies an increased fatty acid uptake into the liver. Both these mechanisms, de novo lipogenesis and hepatic fatty acid uptake, have been shown to contribute to the onset of hepatic steatosis and are enhanced in patients with NAFLD [31]. A distinct daily periodicity has been recognized for various aspects of lipid metabolism, including the expression levels of many hepatic enzymes implicated in lipid biosynthetic pathways [40]. In the liver of rodents, lipogenic gene expression peaks during the dark-time, whereas lipolytic pathways predominate during the light/rest period [41], and these daily oscillations are under the coordinated control of feeding/fasting cycles and the circadian clock [42]. Therefore, we can hypothesize that dLAN exposure can misalign lipid homeostasis through disruption of the circadian-controlled balance between lipogenic and lipolytic pathways in the liver.

De novo synthesis of fatty acids is fueled by nonlipid substrates, such as glucose [5]. Indeed, in dLAN-exposed rats, we found upregulation of the hepatic glucose transporter *Glut2*, which is the main entrance for glucose into the liver. Gene expression of *Glut2* is glucose sensitive, and its regulation couples glucose and lipogenic signaling [43]. In our study, dLAN exposure tended to increase daytime glycaemia, but this effect was inconsistent. Likewise, other studies did not find substantial dLAN effects on basal glycaemia in mice [17,44] and rats [15,16]. On the other hand, preferential utilization of carbohydrates over lipids was demonstrated in mice after a 2-week dLAN exposure [45], and this observation might be consistent with the elevated hepatic glucose uptake and increased fat storage found in our study. Glucose and fatty acid metabolism are connected via several common regulatory mechanisms, among which insulin signaling plays an important role [4]. Consequently, hyperinsulinemia and peripheral insulin resistance are typically associated with hepatic steatosis [3]. Interestingly, we recorded reduced plasma insulin levels after 5 weeks in dLAN, indicating that excessive hepatic TAG accumulation was independent of high insulin. This finding differs from data obtained in spontaneously hypertensive rats, in which dLAN exposure exacerbated already existing hyperinsulinemia [27]. Therefore, we can assume that dLAN may differentially alter metabolic pathways depending on the actual health status.

The adipose tissue plays an important role in maintaining lipid homeostasis. Studies in humans have shown a positive correlation between the expression of lipogenic enzymes in subcutaneous fat and insulin sensitivity, thereby suggesting that lipid deposition in the adipose tissue may function to prevent peripheral lipotoxicity [46]. We found decreased levels of *Fasn* mRNA in the epididymal fat pad of dLAN-exposed rats, indicating diminished de novo lipogenesis and potential negative dLAN effects on the buffering capacity of the adipose tissue. Moreover, dLAN-exposed rats displayed lower plasma leptin and a trend towards higher adiponectin levels compared to controls. Both adipokines are essentially implicated in metabolic and energy homeostasis through their central or peripheral actions, including direct effects on the liver [33]. Leptin has suppressive effects on hepatic de novo lipogenesis and stimulates fatty acid oxidation, providing protection against TAG accumulation in nonadipose tissues and, consequently, from lipotoxicity [47]. Accordingly, hepatic steatosis develops in leptin-deficient animal models [48], and an inverse link between serum leptin and the degree of steatosis was shown in patients with bariatric surgery [49]. Therefore, decreased leptin levels following dLAN exposure could lead to the attenuated antisteatotic action of leptin. However, mechanisms behind the dLAN-induced decrease of circulating leptin levels need to be further investigated in a circadian context. For example, they can relate with a negative energy balance caused by misalignment between timing of food intake and metabolic circadian rhythms, as was hypothesized in the study in humans who underwent circadian misalignment [50].

Mistimed food intake has been proposed as one of the important explanatory factors linking light at night and obesity [51]. Experiments in mice showed that dLAN exposure shifted food intake into the rest period, which in parallel accounted for increased body mass gain [26], while the diminished amplitude in the rhythm of food intake was reported without effects on body mass in rats [16]. In our study, dLAN-induced metabolic effects in the liver and adipose tissue were not reflected in changes in body mass and total daily food intake, although we cannot exclude modification of the timing in food consumptions.

To examine the molecular mechanisms of dLAN-induced metabolic effects, we focused on the PPAR family of nuclear receptors that function as transcription factors in many aspects of lipid and glucose metabolism [6]. We found that 2 weeks of dLAN exposure resulted in upregulated hepatic *Pparα* expression. In the liver, PPARα is an important nutrient sensor, which regulates various processes of fatty acid metabolism in response to the feeding/fasting cycle [52]. Moreover, hepatic expression of *Pparα* shows a distinct daily rhythm, which increases during the daytime [53], and can be directly transcriptionally controlled by a local circadian clock [54]. Indeed, attenuated rhythms of clock gene expression have been documented in the liver of mice exposed to dLAN [17]. Thus, upregulated *Pparα* caused by dLAN could be explained by disturbed daily rhythmicity, although further studies examining the whole 24-h profile of *Pparα* and its downstream genes in dLAN conditions would be required to verify this link. Nevertheless, our results showed that the increased expression levels of hepatic genes involved in fatty acid synthesis in dLAN-exposed rats can be associated with increased *Pparα* expression, since PPARα can directly or indirectly control not only fatty acid oxidation but also lipogenic pathways [52]. Moreover, a deficiency of PPARα has been reported to abolish daily variations of lipogenic genes in the liver in mice [55], whereas PPARα agonists stimulated the rate of hepatic fatty acid synthesis, a response, which was greater during the light phase than during the dark [32].

In the epididymal fat pad, dLAN-exposed rats displayed increased *Pparγ* expression, the other PPAR family member, which complements PPARα in the regulation of lipid metabolism and energy homeostasis. Likewise, *Pparγ* expression is responsive to nutrient intake and the circadian clock [56], indicating that a changed *Pparγ* expression in dLAN-exposed rats could either reflect disturbed circadian rhythms [14,17] or, alternatively, could compensate disturbed metabolic balance. PPARγ is highly expressed in the adipose tissue, where it regulates adipocyte differentiation and fatty acid metabolism, and it improves insulin sensitivity [57]. In addition, PPARγ participates in the production of several adipokines, including adiponectin and leptin [58]. Accordingly, altered levels of these adipokines in the circulation of dLAN-exposed rats can relate to deregulated *Pparγ* expression in the adipose tissue. Importantly, PPARs have been shown to interact or directly transactivate the main components of the molecular clockwork [54,59], indicating that dLAN-induced changes in the expression of these central metabolic regulators could compromise a crosstalk between the metabolism and the circadian clock.

## 4. Materials and Methods

### 4.1. Animals

**Figure A1:**
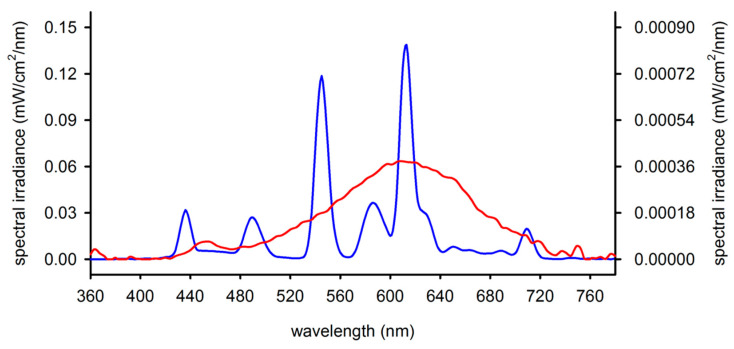
Spectral power distribution of light sources emitted bright white light (150–200 lx) during the light phase (blue line with left y axis) and dim light (~2 lx) during the night phase (red line with right y axis). Spectral power distribution was measured with an illuminance spectrophotometer CL-500A (Konica Minolta Sensing Europe BV, Germany).

Male Wistar rats were obtained at the age of 14 weeks from the breeding station of the Institute of Experimental Pharmacology and Toxicology, Slovak Academy of Sciences (Dobrá Voda, Slovak Republic). The animals were housed in plastic cages in groups of three to four rats at an ambient temperature of 21.5 ± 1.3 °C and a humidity of 55–65%, and they were provided with a standard pelleted diet and water *ad libitum*. During the acclimation period of 4 weeks, rats were maintained on the LD cycle of 12:12 h with lights on at 10:00 a.m., designed as Zeitgeber time 0 (ZT0). At the level of the animal cages, room lights emitted white light with an illumination of 150–200 lx and color temperature of 2900 K (Figure A1).

The experimental procedure was approved by the Ethical Committee for the Care and Use of Laboratory Animals at the Comenius University in Bratislava, Slovak Republic, and the State Veterinary Authority of the Slovak Republic (Ro-1648/19–221/3; approved 14 June 2019).

### 4.2. Experimental Design

After acclimation, rats (311 ± 28 g) were assigned to either the control group (CTRL, *n* = 15) with the standard lighting regime described above or to the experimental group (dLAN, *n* = 18), which was exposed to low-illuminance levels of 2 lx during the entire night phase. The dim light conditions were provided by a shaded lamp with LED bulb Star Classic A60 10W (OSRAM GmbH, Munich, Germany), which emitted a broad-spectrum white light with a peak of 610 nm and color temperature of 2700 K (Figure A1). Illuminance and color temperature were measured at the level of the animal cages using an illuminance spectrophotometer CL-500A (Konica Minolta Sensing Europe BV, Bremen, Germany). 

Body mass and food intake were recorded weekly throughout the experiment, and total body mass gain and daily food intake were calculated per animal.

Following 5 weeks of the experiment, rats (CTRL, *n* = 7; dLAN, *n* = 9) were blood sampled at ZT9 and ZT21 from the tail to evaluate dLAN effects in association with a daily variation in glucose and insulin levels. Rats were immobilized under isoflurane anesthesia, and blood was collected from a lateral tail vein into heparin tubes. Blood was centrifuged (2500 g, 10 min, 4 °C), and the separated plasma was stored at –20 °C until assays were performed.

### 4.3. Tissue Collection

Rats were sacrificed under isoflurane anesthesia during the first half of the light phase (between ZT3 and ZT6) after 2 (CTRL, *n* = 7; dLAN, *n* = 8) and 5 weeks (CTRL, *n* = 8; dLAN, *n* = 10) of the experiment, respectively. Blood was collected into heparin tubes and processed as described above. Epididymal and visceral adipose tissues were dissected and weighed. For hepatic lipid examination and real-time PCR (qPCR) analyses, tissue samples of the liver and epididymal fat pad were immediately frozen in liquid nitrogen and stored at −76 °C.

### 4.4. Hormone Analyses

Plasma levels of insulin and thyroid hormones (T_3_ and T_4_) were determined with a radioimmunoassay (RIA) using a commercial Insulin Rat ^125^I RIA Kit (DRG Instruments GmbH, Marburg, Germany) and T_3_ [^125^I] and T_4_ [^125^I] RIA Kits (Izotop, Budapest, Hungary), according to the manufacturer’s instructions. Plasma leptin and adiponectin levels were measured by an enzyme-linked immunosorbent assay (ELISA) using commercial Rat Leptin ELISA (BioVendor, Brno, Czech Republic) and Rat Adiponectin ELISA Kits (MilliporeSigma, Burlington, MA, USA). Intra-assay variation coefficients were lower than 5% for all assays.

### 4.5. Biochemical Analyses

Total lipids were extracted from the liver (100 mg) using a chloroform/methanol mixture (2:1), according to Folch’s method [60]. The extracts were dried under a stream of nitrogen and reconstituted in isopropanol. Hepatic and plasma TAG levels and concentrations of plasma glucose, total cholesterol, and LDL-cholesterol were quantified enzymatically with commercial kits BIO-LA-TEST (Erba Lachema, Brno, Czech Republic), according to the manufacturer’s instructions, and modified for 96-well plates. Plasma FFAs were determined with a colorimetric assay kit (Cell Biolabs, San Diego, CA, USA).

### 4.6. RNA Isolation and Real-Time PCR

Liver and fat tissue samples were homogenized using a FastPrep instrument (MP Biomedicals, Eschwege, Germany). Total RNA was isolated from the liver with TRI Reagent (Molecular Research Center, Cincinnati, OH, USA) and from the epididymal fat pad with an RNeasy Plus Universal Mini Kit (Qiagen, Hilden, Germany), according to the manufacturer’s instructions. For synthesis of complementary DNA, a Maxima cDNA Synthesis Kit (Thermo Fisher Scientific, Waltham, MA, USA) was used. The quantity and purity of the isolated RNA were measured with a NanoDrop One spectrophotometer (Thermo Fisher Scientific). The RNA integrity was verified with 1.2% agarose gel electrophoresis. Amplification of cDNA was performed with Maxima SYBR Green qPCR Master Mix (Thermo Fisher Scientific) and the CFX Connect real-time PCR detection system (Bio-Rad, Hercules, CA, USA). The relative expression of the target and reference genes was calculated using a standard curve method. The expression of the target genes was normalized to the expression of β-actin (*Actb*) and peptidylprolyl isomerase A (*Ppia*) in the liver and epididymal fat pad, respectively. Primer sequences of target genes are as follows: *Acc* (NM_022193.1), forward 5′-GCA GTC TCC CAA CTC CTA CG-3′, reverse 5′-CAG TCC ACC ATC ACT CAG CC-3′; *Acat* (NM_017075.2), forward 5′-GTC TAC CCA TTG CCA CTC CG-3′, reverse 5′-TGA CAT GCT CTC CAT TCC GC-3′; *Cd36* (NM_031561), forward 5′-ATC GGA ACT GTG GGC TCA TT-3′, reverse 5′-TTC TTC AAG GAC AAC TTC CCT TT-3′; *Fabp4* (NM_053365.1), forward 5′-ATG AAA GAA GTG GGA GTT GGC-3′, reverse 5′-TCT CTG ACC GGA TGA CGA CC-3′; *Noct* (NM_138526.1), forward 5′-CAG TAG CCA TCC TCC CAT TAG-3′, reverse 5′-ATT TCC TCT CCT CCC ATT TGA-3′; *Scd1* (NM_139192.2), forward 5′-AAA CCT GCA GAA TGG ACG AGA-3′, reverse 5′-GGG TCG TGG ATA TCT TCT CTC A-3′. Sequences for other primers were reported previously [27,61].

### 4.7. Statistical Analyses

Statistical analyses were performed using GraphPad Prism v.8 (GraphPad Software, San Diego, CA, USA). All measures were checked for normal distribution with the Kolmogorov–Smirnov test. Data for plasma glucose, adiponectin, thyroid hormones, and logarithmically transformed insulin and leptin were analyzed using two-way analysis of variance (ANOVA) with factors “Regime” and “Week/ZT,” followed by Bonferroni’s multiple comparisons test, if the interaction was or tended to be significant. Differences in body mass and daily food intake over 5 weeks of the experiment were evaluated using two-way repeated measures ANOVA with between-group factor “Regime” and within-group factor “Day.” For other parameters, CTRL and dLAN groups were compared by the Student’s *t*-test or the Mann–Whitney U test, depending on the normal distribution. A *p*-value of <0.05 was considered to be statistically significant. Data are presented as the mean ± standard error of mean (SEM).

## 5. Conclusions

In conclusion, our data showed that chronic exposure to low-intensity light at night resulted in excessive TAG accumulation in the liver through the deregulated balance of lipid biosynthetic pathways. In dLAN-exposed rats, we found upregulated hepatic genes involved in de novo lipogenesis and elevated glucose and fatty acid uptake. These effects were independent of insulin levels that were even reduced in rats after 5 weeks in dLAN. In the adipose tissue, dLAN suppressed fatty acid synthesis and altered circulating levels of leptin and adiponectin. Moreover, dLAN-exposed rats displayed elevated hepatic and adipose tissue expression of important metabolic regulators *Pparα* and *Pparγ*, indicating compromised transcriptional control of lipid homeostasis. No changes were found in body mass, total food intake, and plasma thyroid hormone levels showing that these parameters were not primarily involved in metabolic effects caused by dLAN. Taken together, our results contribute to understanding the molecular mechanisms by which dLAN can affect lipid metabolic homeostasis and can be linked with the development of metabolic diseases.

## Abbreviations

ACAT	Acetyl-CoA acetyltransferase
ACC	Acetyl-CoA carboxylase
ACTB	β-actin
CD36	Fatty acid translocase
CHOL	Cholesterol
CTRL	Control
dLAN	Dim light at night
FABP4	Fatty acid binding protein 4
FASN	Fatty acid synthase
FFA	Free fatty acid
GLUT	Glucose transporter
HMGCS1	3-hydroxy-3-methylglutaryl-CoA synthase 1
HSL	Hormone-sensitive lipase
IRS2	Insulin receptor substrate 2
LD	Light–dark
LDL	Low-density lipoprotein
NAFLD	Nonalcoholic fatty liver disease
NOCT	Nocturnin
PPARα	Peroxisome proliferator-activated receptor alpha
PPARγ	Peroxisome proliferator-activated receptor gamma
PPIA	Peptidylprolyl isomerase A
PYGL	Glycogen phosphorylase L
SCD1	Stearoyl-CoA desaturase 1
T_3_	Triiodothyronine
T_4_	Thyroxine
TAG	Triacylglycerol
ZT	Zeitgeber time
